# Accumulation of metabolic side products might favor the production of ethanol in Pho13 knockout strains

**DOI:** 10.15698/mic2016.10.532

**Published:** 2016-09-23

**Authors:** Guido T. Bommer, Francesca Baldin, Emilie Van Schaftingen

**Affiliations:** 1 Walloon Excellence in Lifesciences and Biotechnology (WELBIO) and Laboratory of Physiological Chemistry, de Duve Institute, Université Catholique de Louvain, 1200 Brussels, Belgium.

**Keywords:** glycolysis, metabolite repair, side product, ethanol formation, xylose, Pho13, phosphoglycolate phosphatase

## INTRODUCTION

Enzymes of intermediary metabolism are very specific. However, they are not absolutely specific, although some biochemistry textbooks might give this impression. They catalyze side reactions at modest, yet significant rates, by acting on substrates that structurally resemble their physiological substrates. The resulting side-products will accumulate and may have detrimental effects (e.g. by inhibiting other enzymes), unless they are metabolized. We and others have found several dedicated enzymes that serve to eliminate metabolic side-products, a process which has been variously named ‘metabolite repair’ or ‘metabolite pool sanitization’ 1. In some instances, mutations in genes involved in these processes lead to disease, underlining the importance of these processes for overall metabolism.

**Figure 1:**
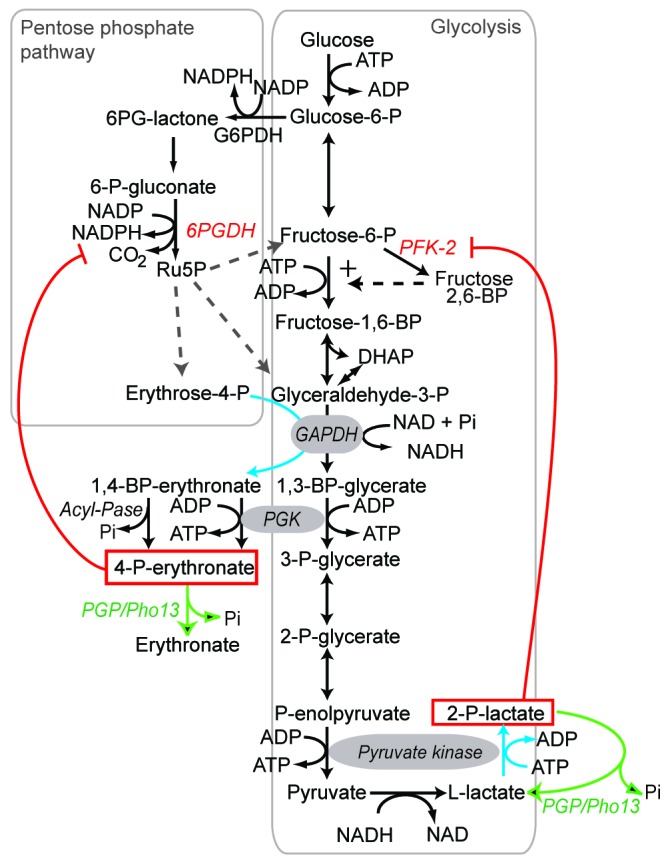
FIGURE 1: The enzyme PGP/Pho13 eliminates glycolytic side products and allows parallel action of glycolysis and the pentose phosphate pathway. Side activities of glycolytic enzymes are shown in blue; new metabolite repair activities in green and inhibitory interferences discovered or characterized in the paper of Collard and colleagues in red. 6PGDH, 6-P-gluconate dehydrogenase; PGK, phosphoglycerate kinase; 6PG-lactone, 6-P-gluconolactone; Ru5P, ribulose-5-P; BP, bisphospho; PFK-2, phosphofructokinase-2. Modified from 2.

In a paper recently published in *Nature Chemical Biology* 2, we describe the discovery of a striking example illustrating the metabolite repair concept. We found that a single phosphatase serves to destroy three side-products of glycolysis, 4-P-erythronate, 2-P-glycolate and L-2-P-lactate (Fig. 1). When starting this work, we were motivated to search for an enzyme destroying 4-P-erythronate, because the literature contained evidence that glyceraldehyde-3-P dehydrogenase acts significantly, though with a low catalytic efficiency, on erythrose-4-P, thereby causing the formation of 4-P-erythronate, a potent inhibitor of 6-P-gluconate dehydrogenase (Ki < 1 µM). We expected that if cells did not have an enzyme - most likely a phosphatase - serving to destroy this compound, 4-P-erythronate would accumulate and inhibit the oxidative part of the pentose phosphate shunt, a major metabolic pathway (Fig. 1). We indeed found a 4-P-erythronate phosphatase in mammalian tissues and identified it as a phosphatase known to destroy P-glycolate, which is accordingly named P-glycolate phosphatase (PGP). Both activities were conserved in its yeast orthologue, Pho13.

Knocking out of PGP in mammalian cells led to the accumulation of 4-P-erythronate to concentrations (30 µM) able to inhibit 6-P-gluconate dehydrogenase. Expectedly, it caused an enormous increase (>100-fold) in the concentration of 6-P-gluconate and of its dephosphorylation product gluconate. It also led to the accumulation - not of 2-P-glycolate - but of a closely related compound, 2-L-P-lactate, a non-classical phosphate ester, which is also an excellent substrate for mammalian PGP. *In vitro* experiments with isotope-labeled metabolites demonstrated that L-2-P-lactate is produced from L-lactate, likely by a slow side activity of pyruvate kinase. Accumulation of L-2-P-lactate contributes to the strong metabolic disturbances in PGP knockout cells. Like its structural analogue P-enolpyruvate, L-2-P-lactate is a powerful inhibitor of some forms of PFK2-FBPase2, the enzyme that produces fructose-2,6-bisphosphate, a major activator of glycolytic flux 3. This presumably explains the low level of fructose-2,6-bisphosphate and low glycolytic flux observed in PGP-KO cells.

Knocking out of Pho13 in yeasts similarly caused accumulation of 4-P-erythronate and an increase in the concentrations of 6-P-gluconate. We did not observe any increase in 2-P-lactate, likely because L-lactate is not present in yeast, but we did observe an increase in 2-P-glycolate, which is presumably formed by phosphorylation of glycolate (a normal metabolite in yeast) by a side activity of pyruvate kinase. Although this has not been further studied in yeast, it is known that P-glycolate is a strong inhibitor of triose-phosphate isomerase and that it is a potent stimulator of the 2,3-biphosphoglycerate phosphatase activity of phosphoglycerate mutase. So, in all likelihood, P-glycolate accumulation may also lead to perturbations of intermediary metabolism. When we compared the metabolic changes in Pho13-deficient yeast to those observed in mammalian PGP KO cells, we noted that the increase in 6-P-gluconate levels in yeast was not as dramatic (8-fold versus >100-fold). The reason for this is that Pho13 KO also induces a several fold increase in the expression of several enzymes of the pentose-phosphate pathway and particularly 6-P-gluconate dehydrogenase and transaldolase (see below), presumably as a response to the metabolic block imposed by the accumulation of 4-P-erythronate (Fig. 1).

**Figure 2:**
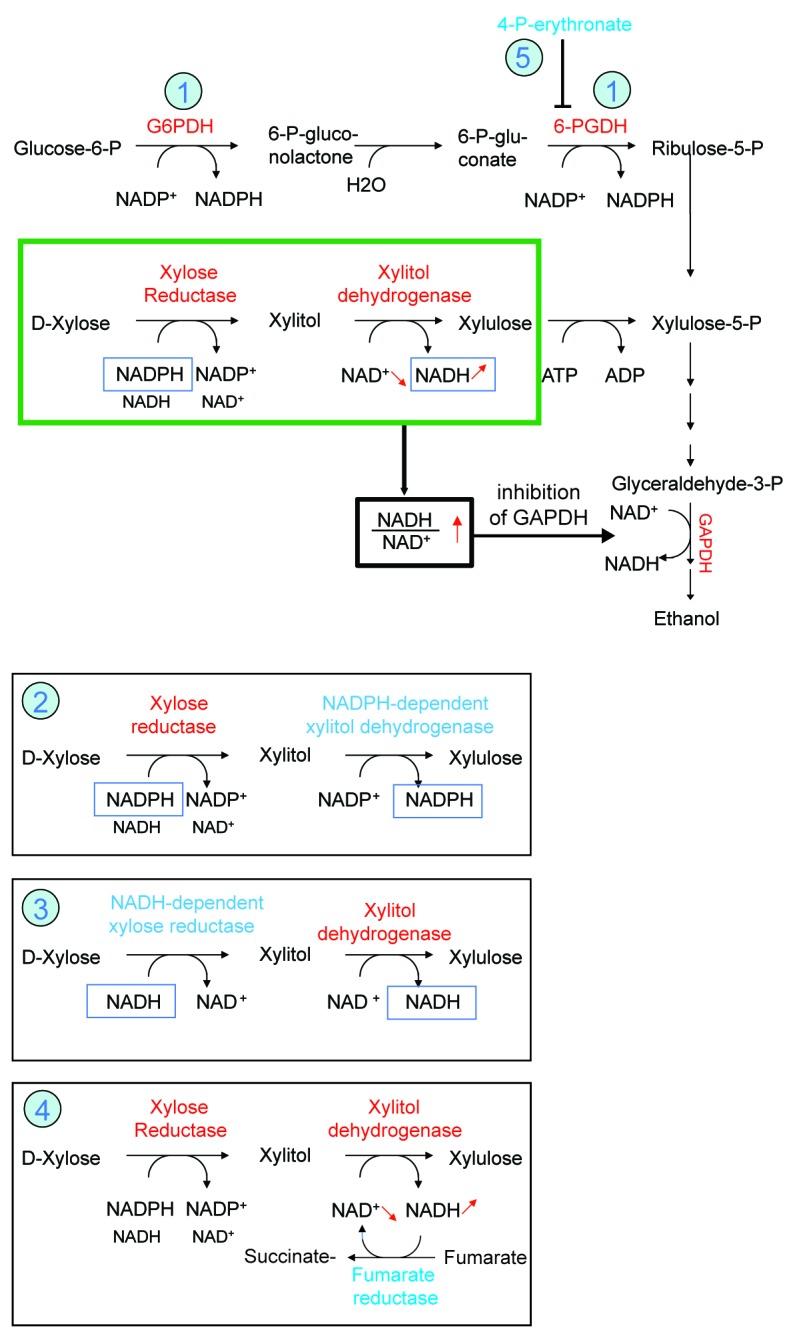
FIGURE 2: Inhibition of the pentose phosphate pathway prevents nicotinamide nucleotide imbalance and facilitates production of ethanol from xylose. Preferential usage of NADPH by xylose reductase and NAD^+^ by xylitol dehydrogenase leads to an increase in cellular NADH/NAD^+^ ratio which limits glyceraldehyde 3-phosphate dehydrogenase (GAPDH) activity (green box). This can be alleviated (highlighted in blue) by **(1)** reducing NADPH production in the oxidative pentose phosphate pathway by deleting the enzymes glucose-6-phosphate dehydrogenase (G6PDH) or 6-phosphogluconate dehydrogenase (6-PGDH), **(2)** by expression of a NADP-dependent xylitol dehydrogenase, **(3)** by expression of a NADH-dependent xylose reductase, **(4)** expression of NADH-consuming fumarate reductase or **(5) **by inactivation of PGP, which leads to the accumulation of 4-P-erythronate leading to an inhibition of 6-PGDH in the pentose phosphate pathway.

The Pho13 gene has received considerable attention in the field of biotechnology, because it has been repeatedly observed that inactivation of Pho13 improves ethanol production in yeasts that have been engineered to metabolize xylose 4,5. Though not mentioned in our paper, the partial block that 4-P-erythronate causes on 6-P-gluconate dehydrogenase in yeasts offers a potential explanation for these observations 4,5,6. These yeasts have been genetically modified to metabolize xylose, in three steps, into xylulose-5-P, a metabolite that can be used in the pentose phosphate pathway. This is achieved by expressing xylose reductase (which uses NADPH or NADH to generate xylitol), xylitol dehydrogenase (which specifically uses NAD^+^ to convert xylitol into xylulose), and xylulose kinase. The cellular NADPH/NADP^+^ ratio is much higher than the cellular NADH/NAD^+^ ratio (Fig. 2). Hence, xylose reductase in the first step preferentially uses NADPH (Fig. 2). Since xylitol dehydrogenase can only use NAD^+^, the combination of the first two reactions leads to a transfer of reducing equivalents from NADPH to NAD^+^ (marked with a green box, Fig. 2) and an increase in the NADH/NAD^+^ ratio, which inhibits glyceraldehyde-3-P dehydrogenase, thereby hampering glycolysis and ethanol formation. This interpretation is supported by the finding that xylose conversion to ethanol may be improved by (**1**) reducing the cellular capacity to synthesize NADPH via a suppression or reduction of the activity of enzymes of the pentose phosphate pathway such as glucose-6-P dehydrogenase, 6-P-gluconate dehydrogenase or phosphoglucose isomerase 7; (**2**) reversing the dependency of xylitol dehydrogenase from NAD^+^ to NADP^+^ 8; (**3**) using a mutated xylose reductase that preferentially uses NADH instead of NADPH 9; (**4**) expressing an NADH-dependent fumarate reductase, which eliminates the excess of NADH production 10.

In this context, the simplest explanation for the beneficial effect of Pho13 deficiency is that it leads to (**5**) an increase in the concentration of 4-P-erythronate. The resulting inhibition of 6PGDH limits the formation of NADPH in the oxidative pentose phosphate pathway and thereby prevents a preferential use of NADPH over NADH in the xylose reductase reaction. In addition to this, the increased expression of transaldolase in Pho13-deficient cells (found both by us and Kim and colleagues 11) also seems to facilitate xylose metabolism 12. Overall, the inactivation of a metabolite repair system that seems to be beneficial to most organisms (as indicated by its high evolutionary conservation) may lead to advantages under very specific circumstances.

A potentially confusing aspect of PGP/Pho13 function is that a single enzyme serves to destroy three distinct metabolites that may perturb intermediary metabolism. Does this mean that PGP/Pho13 is a non-specific phosphatase? Certainly not, since it does not display significant activity (catalytic efficiency more than 50-fold lower) on several classical metabolites (2-P-glycerate, 3-P-glycerate) despite their close structural similarity to the three best substrates (4-P-erythronate, L-2-P-lactate, 2-P-glycolate). This kind of ‘negative specificity’ is impressive and may turn out to be a hallmark of other enzymes active in metabolite repair.

Mammalian PGP and yeast Pho13 have been known for decades. Yet their most striking function was only revealed as a result of hypothesis-driven search for an enzyme that destroys metabolites that are expected to exist, but have not been described before. Many metabolic side-products are likely absent from conventional metabolite libraries and some of them may have detrimental effects even at very low concentrations (as exemplified by the case of 4-P-erythronate). This makes these side-products very difficult to identify in untargeted metabolomics studies. Therefore, the identification of PGP/Pho13 as 4-P-erythronate phosphatase highlights the importance of hypothesis-driven research targeted towards non-canonical metabolites, if we want to understand the function of remaining orphan enzymes.
